# Bibliometric Analysis of Vitamin D Research 2019-2024

**DOI:** 10.7759/cureus.96510

**Published:** 2025-11-10

**Authors:** Devin F Hannon, Syed M Shah, Camille Moeckel, Mudasir Umer, Erum Azhar, Abdul Waheed

**Affiliations:** 1 Internal Medicine, Creighton University School of Medicine, Phoenix, USA; 2 Internal Medicine, Wright Center Internal Medicine Residency Program, Scranton, USA; 3 Family Medicine, Penn State University College of Medicine, Milton S. Hershey Medical Center, Hershey, USA; 4 Family Medicine, International University of Kyrgyzstan, Bishkek, KGZ; 5 Obstetrics and Gynecology, Dignity Health East Valley Obstetrics and Gynecology Residency Program, Gilbert, USA; 6 Obstetrics and Gynecology, Creighton University School of Medicine, Phoenix, USA; 7 Family and Community Medicine, Creighton University School of Medicine, Phoenix, USA; 8 Family Medicine, Dignity Health Medical Group, Gilbert, USA

**Keywords:** bibliography, research funding, trend analysis, vitamin-d, vitamin-d deficiency

## Abstract

Vitamin D research is an important area of biomedical research. This study is a bibliometric analysis that investigated trends in vitamin D research since 2019. A bibliometric analysis was conducted using the SCOPUS database for data extraction. A total of 10,449 articles (2019-2024) were analyzed using the Biblioshiny package in RStudio. Articles were included if they contained "Vitamin D" in titles, abstracts, and keywords. The highest scientific production on vitamin D was observed in the USA (5,354), followed by China (4,842), which showed a steep increase in research output over time. Both had more single-country than multi-country publications, with international co-authorship at 21.02% of publications. Between the peak in 2021 (1,895 articles) and the low in 2024 (1,584 articles), vitamin D research output declined by 16.52%; citation rates also decreased. The *Nutrients* journal saw a steep increase in publications starting in 2021. The most cited study was identified, and its content was reviewed. Prevalent research topics included epidemiology, drug therapy, and prevention and control. Despite early momentum, vitamin D research output and citations have declined in recent years, indicating a decreasing interest in the field. The imbalance between single- and multi-country authorship suggests an opportunity for greater global collaboration. Future work may benefit from interdisciplinary partnerships across institutions.

## Introduction and background

Vitamin D, once primarily recognized for its role in bone health, has recently gained attention for its potential involvement in various chronic diseases [1-3]. In the early 2000s, emerging research highlighted its involvement in conditions such as diabetes, obesity, cardiovascular disease, osteoporosis, mild cognitive impairment, and depression. Daley et al. [4] investigated studies that indicate that vitamin D deficiency increases the risk of type 1, type 2, and gestational diabetes mellitus. An inverse relationship exists between vitamin D status and obesity across diverse populations and ages [5], possibly due to its effects on adipocyte metabolism. Furthermore, studies suggest that vitamin D levels are inversely correlated with risk of cardiovascular disease [6,7]. Vitamin D deficiency can also lead to secondary hyperparathyroidism, which causes increased bone turnover, increased risk of osteomalacia and osteoporosis, and increased risk of fracture [8]. Neurologically, a meta-analysis by Zeqaj et al. [9] suggests that vitamin D supplementation may improve outcomes in patients with mild cognitive impairment, though its utility in Alzheimer's dementia may be limited. Finally, some meta-analyses even correlate low levels of vitamin D with increased symptoms of depression, though other meta-analyses had conflicting results [10,11]. Thus, the study of vitamin D has expanded beyond its traditional role in calcium metabolism and musculoskeletal health.

Considering the extensive impact of vitamin D on health, as well as prior studies evaluating trends in vitamin D research and funding, it is crucial to examine how the vitamin D research landscape has evolved in recent years. Yang et al. [3] found in 2019 that vitamin D research output increased most rapidly from 2008 to 2013 and then plateaued around 2015. Meanwhile, in 2017, Chambers et al. [12] found that the proportion of federal research funding dollars on all vitamins in 2015 was less than a third of what it was in 1992. Umer and colleagues recently published data on publication and funding trends for vitamin D over the last two decades [13]. It found that NIH funding to vitamin D research significantly predicted the total output of vitamin D research. Additionally, Umer et al.'s data show a decreasing trend in vitamin D publications since 2021, despite decades of increasing publications in the years prior. The objective of this study is to explore trends in vitamin D-related research from 2019 to 2024 through an in-depth bibliometric analysis. This study period was chosen to capture the period of transition and better understand the observed shift.

## Review

Materials and methods

Scopus Data Collection

This study is a bibliometric analysis of vitamin D-related literature from January 01, 2019, to December 31, 2024, using Preferred Reporting Items for Systematic Reviews and Meta-Analyses (PRISMA) guidelines [14]. The literature was accessed on January 31, 2025. The literature search was performed on SCOPUS to analyze both quantitative and qualitative trends in vitamin D-related research during the study period. The inclusion criteria for the literature search were journal articles written in English that included "vitamin D" or "vitamin-D" in the keywords, title, or abstract. The search excluded articles related to engineering, computer science, and veterinary topics. This search yielded 10,449 articles for analysis.

**Figure 1:**
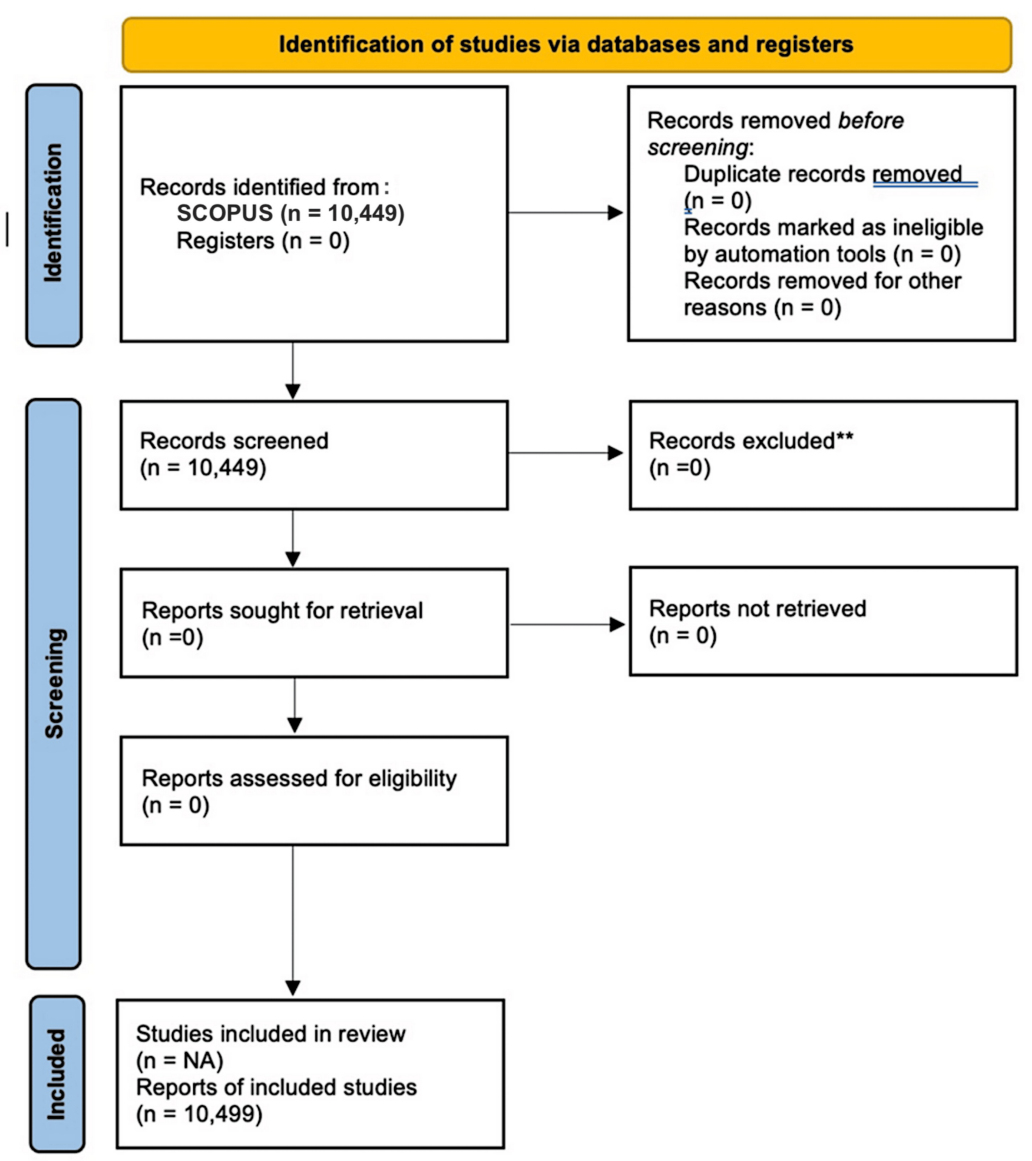
PRISMA Flow Diagram of Data Collection for Bibliometry PRISMA: Preferred Reporting Items for Systematic Reviews and Meta-Analyses

Figure 1 below shows the PRISMA flow of the literature search and selection. Of note, the articles were not sought for retrieval because analysis of their study methods, results, or conclusions was beyond the scope of this study. Furthermore, because the inclusion criteria consisted of journal articles exclusively, the number of studies included in the review was not available. For bibliometric analyses, the PRISMA flow diagram can sometimes appear nontraditional when compared to systematic reviews or meta-analyses.

Quantitative data and citation metrics were analyzed, including the annual volume of vitamin D-related publications and research contributions from different countries, institutions, and journals. Additionally, qualitative trends, including shifts in research impact based on publication and citation patterns and the scope of international collaborations, were analyzed to evaluate changes in the field's focus and identify future directions for vitamin D research.

The study posed no ethical considerations. It did not involve patient care data, and no direct patient information was accessed. Thus, no consenting of subjects was involved either. The Institutional Review Board (IRB) for the Human Subjects Protection Program (HSRP) at Penn State College of Medicine, Hershey, deemed this study exempt from review. The IRB classified the study as Non-Human Subjects Research (NHSR) through a notification under Study ID STUDY00025684.

Data Analysis

Our bibliometric analysis encompassed both general and specific parameters, including countries/regions, authors/institutions, journals, documents/references, keywords, and research trends. The collected data were analyzed using the Biblioshiny package in RStudio [15], which provided descriptive metrics across multiple dimensions. Data were then imported into Google Sheets for synthesis, interpretation, and visualization.

This study investigated the worldwide vitamin D research production during the study period. In an effort to analyze the data concisely, the entities that were most successful at producing vitamin D-related research were studied. More specifically, the trends of the five countries that produced the most vitamin D-related research since 2019 were depicted. Additionally, the trends of the five most prominent affiliations and sources were depicted. Lastly, trends in citations of vitamin D research were examined to assess the scientific community's overall interest in vitamin D over recent years.

Results

**Figure 2:**
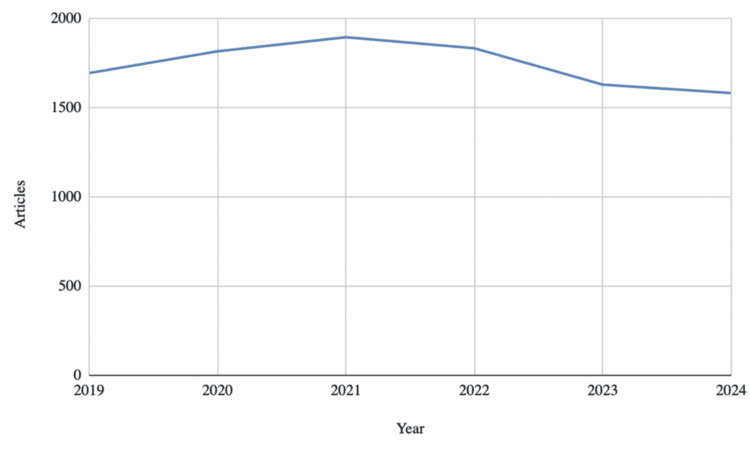
Annual Vitamin D-Related Research

Annual vitamin D research output peaked in 2021 at 1,895 articles published worldwide, but has decreased each year since then (Figure 2). The lowest annual output was 1,582 articles published in 2024, which represented a 16.52% decline from the 2021 peak.

**Figure 3:**
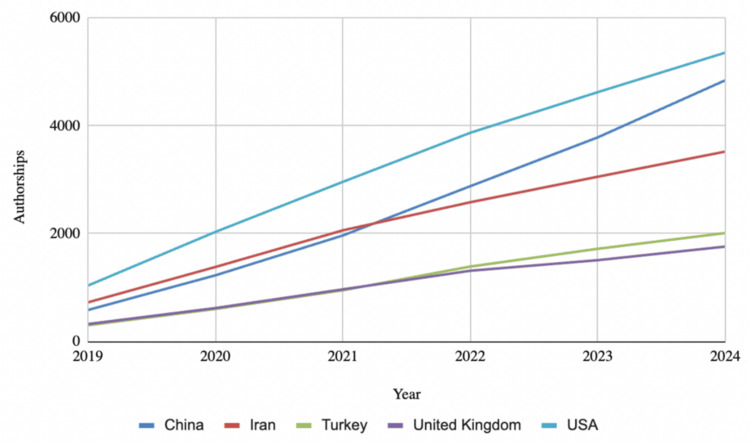
Total Country Production Over Time Since 2019

**Figure 4:**
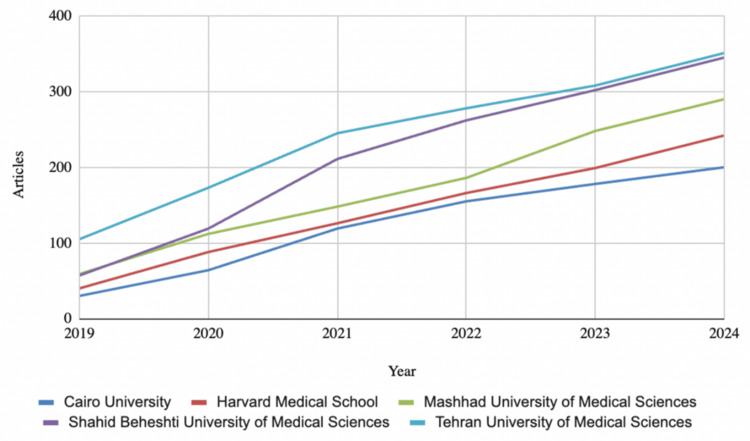
Total Affiliation Production Over Time Since 2019

Since 2019, the United States (U.S.) has led global production with 5,354 authorships overall, followed by China (4,842), Iran (3,520), and Turkey (2,010). Notably, while the U.S. and Iran saw a decline in production over time, China experienced a steady increase in production during this period, with 643 authorships in 2020 and 1058 authorships in 2024 (Figure 3). Tehran University of Medical Sciences was the top publisher, contributing 351 articles, followed closely by Shahid Beheshti University in Iran with 345, Mashhad University in Iran with 290, Harvard University in the U.S. with 242, and Cairo University with 200 articles (Figure 4). International co-authorships accounted for just 21.02% of vitamin D research output since 2019.

**Figure 5:**
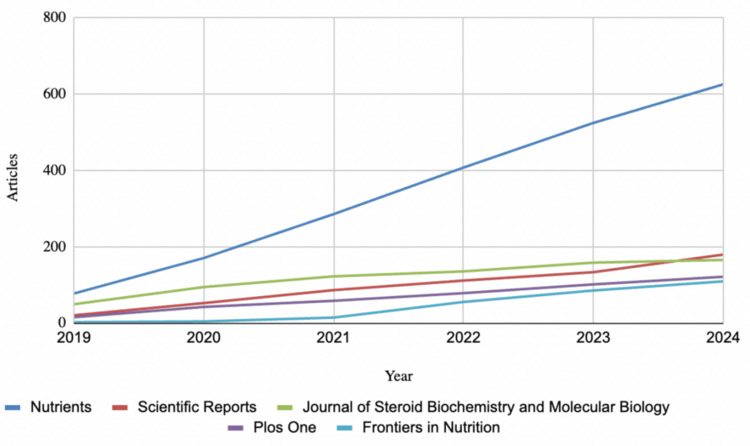
Total Source Production Over Time Since 2019

**Figure 6:**
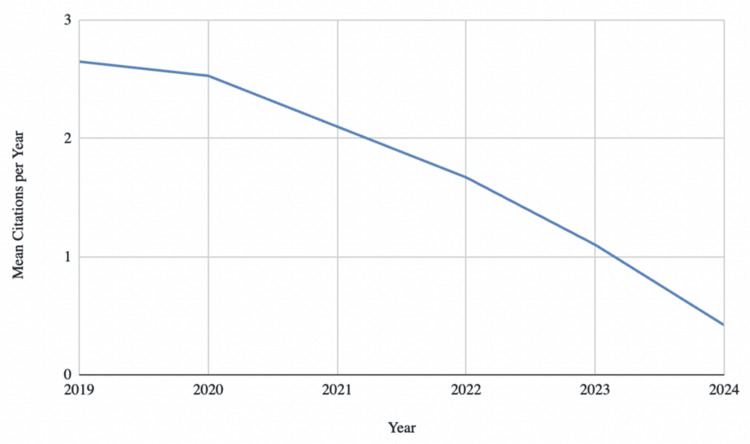
Average Number of Citations Per Citable Year

The most productive journal source was Nutrients, which published 625 articles, followed by Scientific Reports with 179, Journal of Steroid Biochemistry and Molecular Biology with 165, PLOS One with 121, and Frontiers in Nutrition with 109 articles (Figure 5). Notably, Nutrients saw a significant increase in its publication rate starting in 2021, increasing its average annual rate from 85 articles per year in 2019 and 2020 to about 114 articles per year from 2021 to 2024. Figure 6 shows a consistent decline in average citations per article per citable year from 2.65 in 2019 to 1.1 in 2023. The New England Journal of Medicine had the most cited study, authored by Manson et al. in 2019 [16], which focused on vitamin D's role in cancer and cardiovascular disease (1,008 citations).

Discussion

This analysis of vitamin D-related research trends from 2019 to 2024 reveals several shifts in the field. While the early 2020s witnessed a surge in research output, the data suggest that interest in vitamin D research has waned since its peak in 2021. While no P-value was obtained in this study, this decline represents the turning point after the substantial surge in vitamin D research output that took place during the 2010s. This decline may reflect a decreasing interest in the field or a shift in the focus of scientific research towards emerging health concerns, such as the COVID-19 pandemic. The pandemic disrupted scientific research through many mechanisms. As reported by Sohrabi et al. [17], the pandemic led to widespread laboratory closures, a shift in research focus toward urgent COVID-related topics, and deployment of researchers to clinical duties. All of these immediate reactions during that period may have negatively affected vitamin D research output. While COVID-19 still causes significant health challenges globally, it will be important for the research community to resume prior efforts on preventative medicine, including vitamin D-related topics.

The decrease in the average number of citations per article per citable year further supports the hypothesis that the attention and impact of individual vitamin D-related studies have decreased over time. In general, when new research is published, citation rates are usually highest in the years immediately after it is released. This is because subsequent researchers prefer to cite the newest, most up-to-date research. Thus, one typically expects the citation rate per citable year to be highest in the year following publication. However, the findings of this study indicate that the trend for this metric with regard to vitamin D research is just the opposite. That is, the research published in 2019 has been cited the most per citable year, and the citation rates of the most recent studies have decreased substantially. The average number of citations for the studies published in 2023 was only 1.1 per year. Meanwhile, the studies published in 2019 were cited an average of 2.65 times per year, despite being older.

One potential explanation for the heightened interest in older studies compared to newer ones is the extensive public media coverage of vitamin D in the 2010s. Caulfield et al. [18] showed that from 2009 to 2014, the media (newspapers, TV coverage) reported high rates of favorable opinions of vitamin D supplementation. When pairing this data with the fact that vitamin D research accelerated in 2013, it is reasonable to hypothesize that the media affects research output. However, there is a lack of recent data on media coverage of vitamin D, so this explanation for the study period is chronologically distant. Nevertheless, this concept provides an opportunity for further research on this topic, especially given the evolution of social media and the growing popularity of health influencers.

The geographical analysis of publication output highlights a concentration of research activity in a few countries, with the U.S., China, and Iran contributing the majority of publications. Interestingly, while the U.S. and Iran have experienced a decline in production between 2019 and 2024, China's research output has steadily increased. One explanation for the decreasing output in the U.S. might be the decrease in federal funding. Umer et al.'s data [13] suggest that NIH funding toward vitamin D research has decreased consistently since 2013 and that the amount of funding does significantly predict vitamin D research output. Thus, it is possible that the U.S. and Iran have differing levels of funding and institutional support when compared to China. In addition, the dominance of a few research institutions, particularly those in Iran, emphasizes the regional concentration of expertise and suggests that the field could benefit from broader participation by researchers from other regions, especially in light of the relatively low rate of international co-authorship (21.02%) [19].

One limitation of this study was that the articles were not sought for retrieval. Their study methods, results, and conclusions were beyond the scope of this study, but such information could have been used for deeper qualitative analysis. Other limitations of the study include a lack of statistical analysis and a lack of control for citation lag time bias.

## Conclusions

In conclusion, vitamin D-related research appears to be experiencing a decline in both publication volume and citation impact, suggesting a shift in research focus. The current decline in research output and relatively low international collaboration could provide an opportunity to reinvigorate the field. To maintain the relevance and impact of vitamin D-related research, future work should focus on emerging health concerns. As previously mentioned, numerous correlations have been documented between vitamin D levels and various diseases; however, questions regarding causation remain unresolved. Further research that provides answers to such questions could translate to evidence-based recommendations, such as vitamin D supplementation or other actionable interventions for patients. Further research should also foster greater international collaboration to ensure that vitamin D-related research evolves to address global health challenges.
